# Parathyroidectomy for solitary parathyroid adenoma via trans‐areola single site endoscopic approach: Results of a case‐match study

**DOI:** 10.1002/cam4.7290

**Published:** 2024-05-21

**Authors:** Ling Zhan, Hao Ding, Qiwu Zhao, Jinyue Liu, Juyong Liang, Ming Xuan, Jie Kuang, Jiqi Yan, Lingxie Chen, Wei Cai, Weihua Qiu

**Affiliations:** ^1^ Department of General Surgery, Ruijin Hospital Shanghai Jiao Tong University School of Medicine Shanghai China; ^2^ Department of General Surgery, Ruijin Hospital Gubei Campus Shanghai Jiao Tong University School of Medicine Shanghai China

**Keywords:** 3D virtual modeling, endoscopic parathyroidectomy, primary hyperparathyroidism, single‐site approach

## Abstract

**Background:**

This study aimed to establish the standardized procedure of trans‐areola single site endoscopic parathyroidectomy (TASSEP), and to compare the performance of TASSEP with that of conventional open parathyroidectomy (COP).

**Methods:**

This study enrolled 40 patients with primary hyperparathyroidism (PHPT) who underwent TASSEP, and included 40 of 176 PHPT patients who underwent COP based on propensity score matching. The retrospective analysis was conducted based on prospectively collected data. Perioperative outcomes, including surgical profile, surgical burden and cosmetic results and follow‐up were reported. The learning curve was described using a cumulative sum (CUSUM) analysis.

**Results:**

40 TASSEPs were completed successfully without conversions or severe complications. There was no statistically significant difference in operation time between TASSEP and COP groups (80.83 ± 11.95 vs. 76.95 ± 7.30 min, *p* = 0.084). Experience of 17 cases was necessitated to reach the learning curve of TASSEP. Postoperative pain score and traumatic index (C‐reactive protein and erythrocyte sedimentation rate) in TASSEP were apparently lower than those in COP group (*p* < 0.05). During the proliferation and stabilization phases, TASSEP was associated with significantly better incision recovery and cosmetic scores. Postoperative serum calcium and PTH levels throughout the follow‐up period indicated satisfactory surgical qualities in both groups.

**Conclusion:**

Based on precise preoperative localization and intraoperative planning facilitated by three‐dimensional (3D) virtual modeling, TASSEP can be feasibly performed on selected patients with satisfactory success rates and low complication rates, providing preferable cosmetic results and alleviating the surgical burden to a certain extent.

## Graphical abstract

Based on precise preoperative localization and intraoperative planning facilitated by three‐dimensional (3D) virtual modeling, trans‐areola single site endoscopic parathyroidectomy can be feasibly performed on selected patients with satisfactory success rates and low complication rates, providing preferable cosmetic results and alleviating the surgical burden to a certain extent.

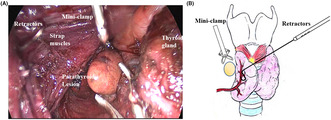

## 1 INTRODUCTION

Primary hyperparathyroidism (PHPT) is a prevalent endocrine disorder characterized by hypersecretion of parathyroid hormone (PTH), with the third prevalence to diabetes and thyroid disease.^1^, ^2^, ^3^ Conventional open parathyroidectomy (COP) with bilateral neck exploration used to be a classic standardized treatment for PHPT with reliable radical outcome.^4^, ^5^, ^6^, ^7^, ^8^, ^9^ Since approximately 85% of PHPT was caused by a solitary parathyroid adenoma,^10^, ^11^ along with the inception of minimally invasive surgery,^12^, ^13^, ^14^, ^15^, ^16^, ^17^, ^18^ focused exploration has been proposed to avoid unnecessary extensive exploration.^19^, ^20^, ^21^, ^22^ Focused parathyroid surgery highlighted the significance of precise preoperative localization.^7^ Besides the high‐resolution ultrasonography, ^99m^Tc‐sestamibi and computed tomography (CT), three‐dimensional (3D) virtual imaging was applied to customize individualized virtual models with specific anatomical details, improving preoperative localization and intraoperative navigation.^16^, ^23^, ^24^ Hence, combing 3D virtual modeling could improve the performance of parathyroid surgery.

However, open parathyroid surgery inevitably came with a conspicuous scar in the anterior neck area and suboptimal cosmesis.^7^, ^8^, ^9^, ^25^, ^26^ Since the first report of transcervical total endoscopic parathyroidectomy in 1996,^27^ parathyroidectomy via endoscopic approach has been unremittingly attempted in pursuing for improved cosmetic expectations, less postoperative pain, and enhanced recovery.^18^, ^20^, ^21^, ^28^, ^29^ On the other hand, there remained controversy over whether multiple‐site endoscopic approach might overlook the potential higher surgical burden to obtain limited cosmetic effects. In this regard, single‐site endoscopic techniques were initiated to further minimize the invasiveness of surgical interventions. Based on previous studies, trans‐areola single site endoscopic technique has validated its feasibility and safety in thyroid disease with superior cosmetic results.^30^, ^31^ However, due to technical challenges and relatively prolonged learning curve, trans‐areola single site endoscopic parathyroidectomy (TASSEP) has only been proposed and illustrated in a few reports with limited cases.^32^ Therefore, in this case‐matched study, we aimed to compare the clinical outcomes of standardized TASSEP with COP, and to evaluate 3D virtual imaging's role in the performance of parathyroid surgery.

## 2 METHODS

### 2.1 Patient inclusion and exclusion

Eligible patients with PHPT were enrolled in study under the following inclusion criteria: (1) a solitary parathyroid adenoma localized unequivocally based on preoperative examination; (2) no evidence of nodular thyroid disease. The exclusion criteria were as: (1) multiple adenomas; (2) equivocal preoperative localization; (3) malignancy; (4) ectopic parathyroid localization; (5) coexisting nodular goiter; (6) familial hyperparathyroidism; (7) history of neck surgery or radiation;

### 2.2 Patient recruitment

**FIGURE 1:**
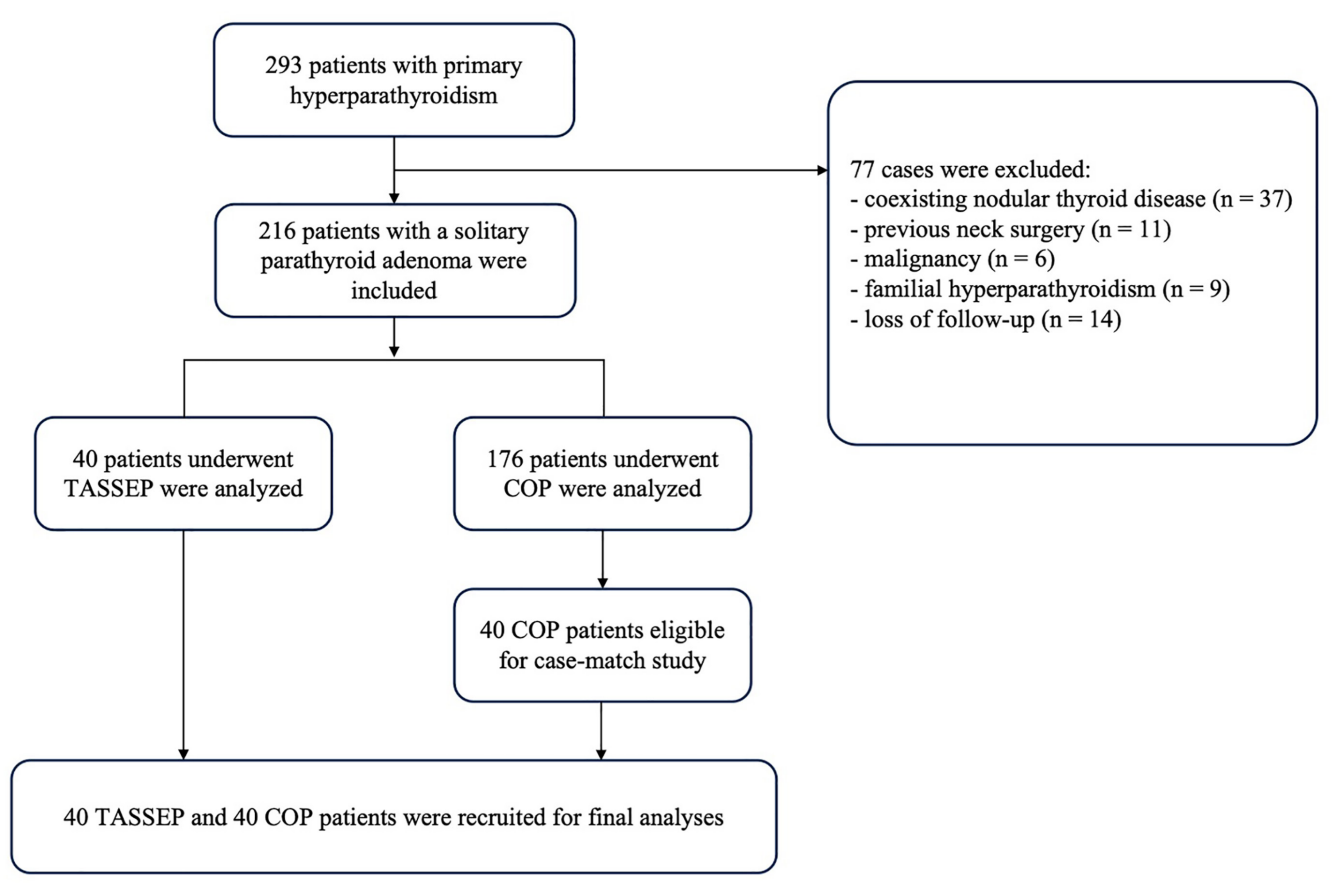
Flow diagram of the study, summarizing a retrospective case‐match study of TASSEP and COP for primary hyperparathyroidism (PHPT). TASSEP, trans‐areola single site parathyroidectomy; COP, conventional open parathyroidectomy.

Between April 2017 and December 2021, a total of 293 patients verified with PHPT were admitted to our center for parathyroidectomy. Of these, 216 patients met the inclusion criteria and 77 patients were excluded on account of coexisting nodular thyroid disease (*n* = 37), previous neck surgery (*n* = 11), malignancy (*n* = 6), familial hyperparathyroidism (*n* = 9), and loss of follow‐up (*n* = 14). All enrolled patients were well‐informed about the TASSEP and COP procedures, and were fully briefed on the potential benefits and risks. Forty of these exhibited strong cosmetic concerns or tended to have scar diathesis underwent parathyroidectomy via trans‐areola single site endoscopic approach. The remaining 176 patients were allocated to the COP group. All surgeries were carried out by the same group of surgeons. This study gained approval from the Ethics Committee and the Institutional Review Board of Shanghai Ruijin Hospital (Ruijin LL‐14‐2006). This work was aligned with the STROCSS standard,^33^ and has been registered at http://www.researchregistry.com (UIN: researchregistry5132). The informed consent form was signed by all patients as agreement to surgery and the use of the data collected during the perioperative period. The flowchart of the study design was summarized in Figure 1.

### 2.3 Matching

To minimize the selection bias when comparing the two different scenarios in this retrospective cohort study, 1:1 propensity score matching was complementarily applied through logistic regression modeling.^34^ A prospectively maintained database containing information on patient and lesion characteristics as well as perioperative records was retrieved to implement the case‐match analysis in this study.^35^, ^36^, ^37^, ^38^ Age, gender, body mass index (BMI), lesion position, lesion size, clinical presentation and values of preoperative serum calcium, phosphorus and PTH were chosen as covariates. Finally, 40 from 176 patients who underwent COP were paired with 40 patients treated with TASSEP, and both groups served as the investigation subjects.

### 2.4 Demographic features

Serum levels of calcium, phosphorus and PTH, and other routine clinical examination were assessed in all cases. High‐resolution ultrasonography, CT, and ^99m^Tc‐sestamibi were carried out in all cases for preoperative localization.

**FIGURE 2:**
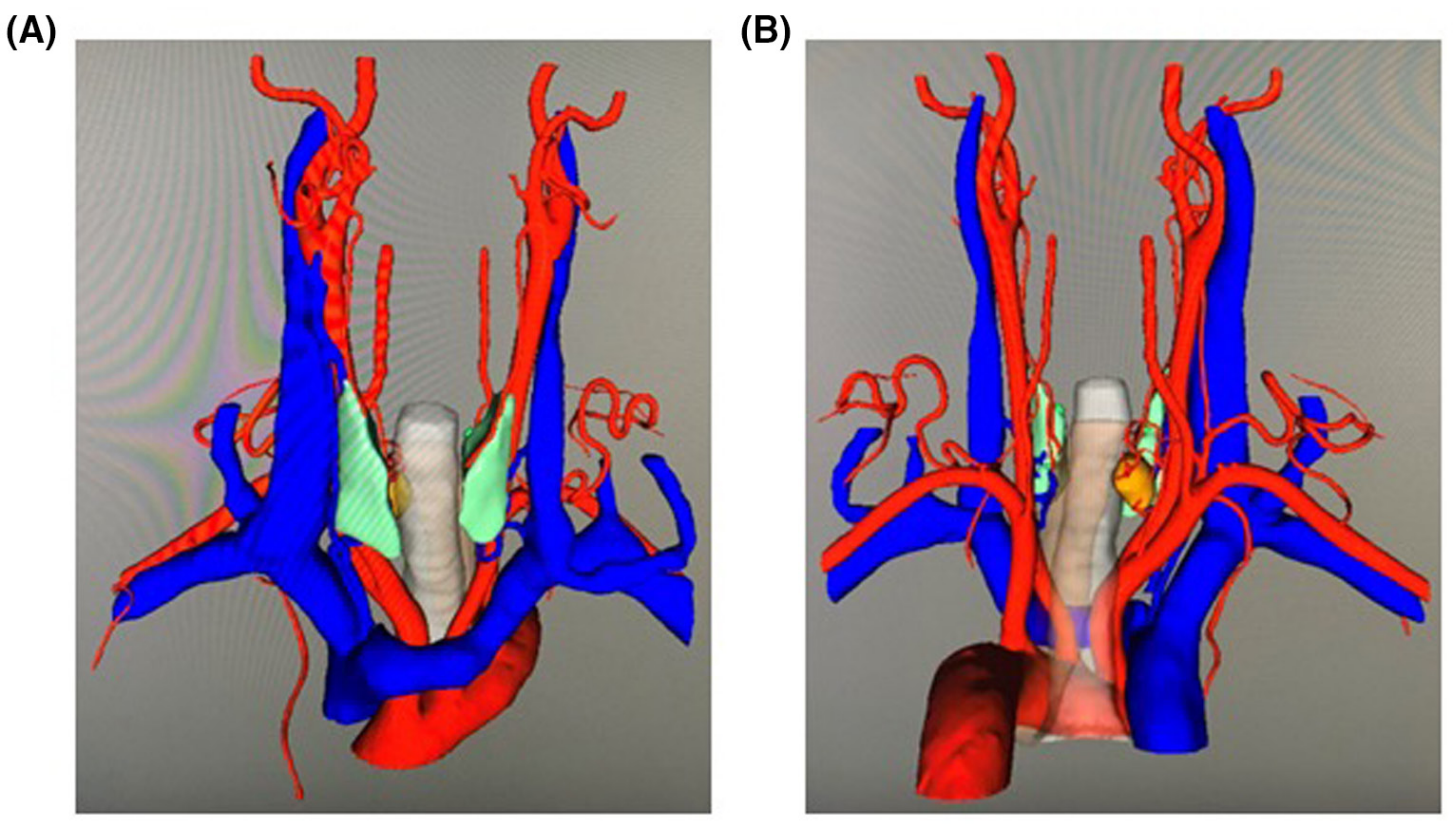
Three‐dimensional (3D) modeling of a lower right parathyroid adenoma. (A) Front view. The 3D modeling optimized visualization and provided positional anatomical details. The lower right parathyroid adenoma was depicted in yellow, thyroid in green, arteries in red and veins in blue. (B) Posterior view.

Film interpretation combined with 3D modeling could illustrate the contour of parathyroid lesions and surrounding infiltrations more accurately.^16^, ^39^ In our study, CT scanning was imported into INCOOL 3D‐Neck Precision Surgical Planning Analysis System (INCOOL Medical Technology, Hangzhou, Zhejiang, China). Lesions were then automatically recognized and the surrounding details were reconstructed by INCOOL 3D‐Neck System at workstation. The morphology profile was recorded and the positional relationship between lesions and the carotid artery and vein, middle thyroid vein, thyroid superior and inferior vessels were defined (Figure 2). By 3D demonstration, the surgical approach and resection plan were finalized individually with visual optimization. Precise surgery planning was completed in coordination with the radiologists and surgeons. Based on above inspections, all patients were identified with the presence of a solitary parathyroid adenoma to an unequivocal location. Additionally, indirect laryngoscopy was applied on admission which confirmed the absence of fixed vocal cord in all patients.

Adhering to the ERAS tubeless principle,^40^ drainage was routinely avoided in both groups. After the operation, postoperative analgesics (tramadol 60 mg, flurbiprofen 300 mg in 100 mL saline solution) were given at 1.5–2.0 mL per hour via an elastomeric pump. Additionally, calcium supplementation was administered based on clinical presentation of hypocalcemia and postoperative serum calcium.

### 2.5 Surgical procedures: TASSEP and COP

**FIGURE 3:**
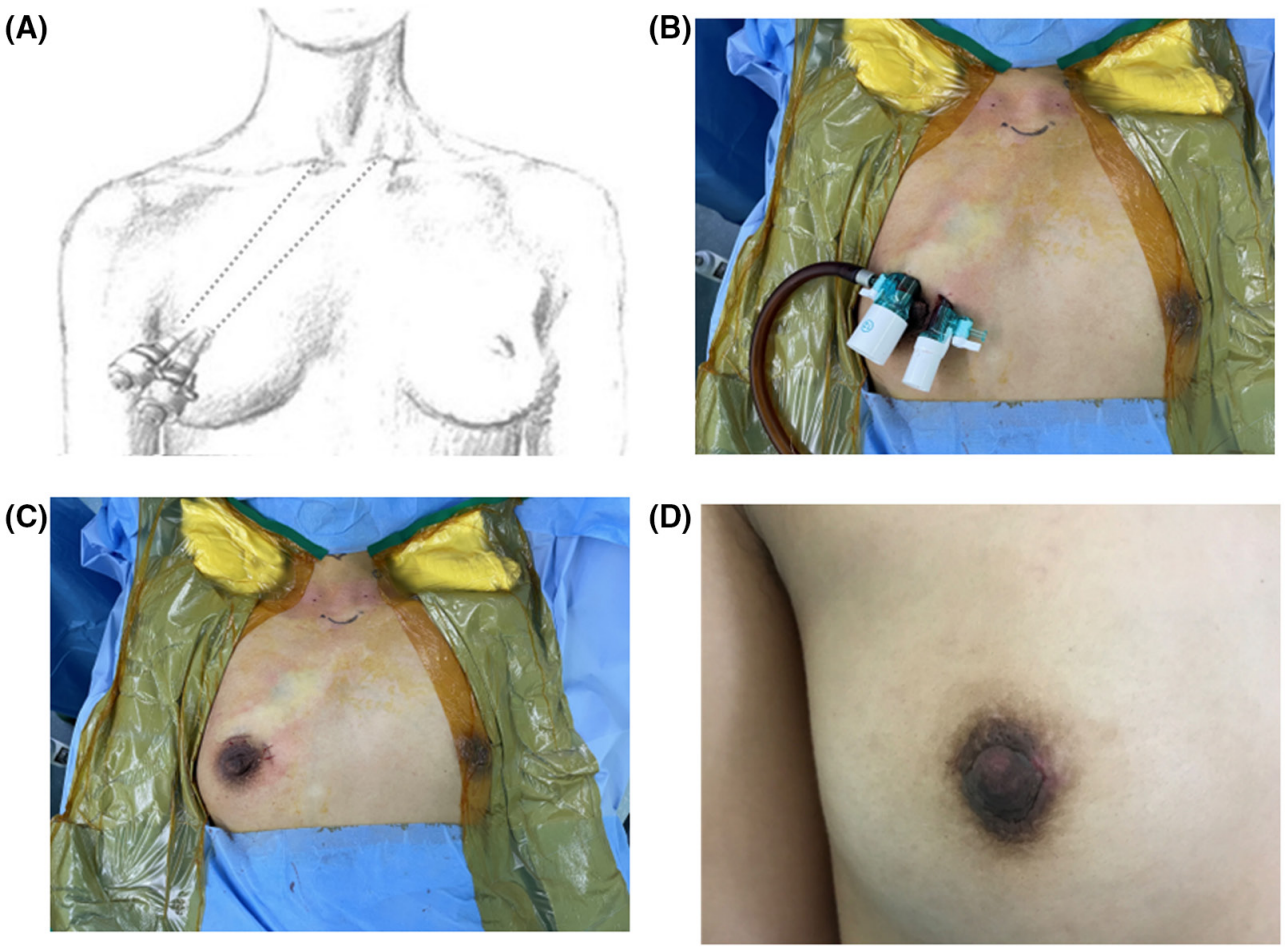
Surgical approach via trans‐areola single site endoscopic parathyroidectomy (TASSEP). (A) The schematic diagram of TASSEP approach. (B) In the areola contralateral to the lesion, one 5‐mm incision and one 12‐mm incision were made at 2 o'clock and 11 o'clock positions, respectively. The placement of trocars during surgery. (C) Post‐surgical appearance of the incisions. Drainage was routinely avoided. (D) The recovery of the incisions 3 months after TASSEP.

**FIGURE 4:**
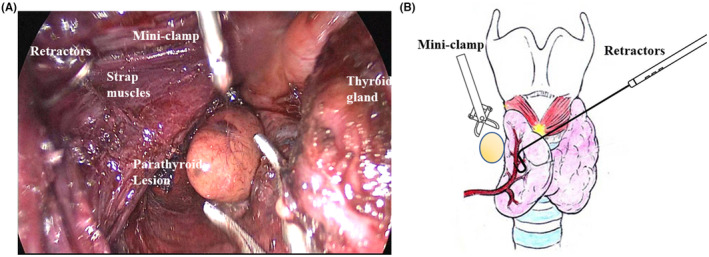
The identification and dissection of parathyroid lesion. (A) Lesion dissection was made possible by means of mini‐clamp (diameter 2.3 mm) and retractors (diameter 1.5 mm). The mini‐clamp reached the surgical field percutaneously, and lesion could be clasped and separated by ultracision scalpel. (B) The scheme of lesion dissection by using retractors and mini‐clamp.

The standardized surgical process for TASSEP is outlined as follows. In the areola contralateral to the lesion, one 5‐mm incision and one 12‐mm incision were made at 2 o'clock and 11 o'clock positions, respectively, which could be camouflaged in natural areola area (Figure 3). Then, 20 mL of tumescence were infused between the deep and superficial layers of the superficial fascia anterior to the manubrium. One surgical apparatus equipped with an Ultracision harmonic scalpel (Harmonic ACE, HAR23; Ethicon Endo‐Surgery, LLC) and one facedown 30° endoscope was inserted into the 5‐mm and 12‐mm trocars, respectively. The outer borders of both sides of the sternocleidomastoid and the upper edge of thyroid cartilage constituted the working space. The needle retractors (diameter 1.5 mm, patented), which accessed the workspace by piercing the neck skin, laterally retracted the anterior and strap muscles, and expanded the interval between the thyroid gland and the strap muscle. Guided by 3D visualization model, both the lesion and the ipsilateral parathyroid were identified. After exposing the RLN at the top of the triangle formed by the inferior thyroid artery and the tracheoesophageal groove, the lesion was lifted using one mini‐clamp (diameter 2.3 mm, patented), fully revealing the lesion boundary (Figure 4). Specimen tissues were dissected with intact capsule, and were then inserted into retrieval bag. After careful hemostasis, the incisions were cosmetically closed with skin glue.

COP was performed as below.^25^ Briefly, a standard collar incision was made. The strap muscles were separated along the midline but not divided laterally, and after mobilizing the thyroid lobe, the exploration was carried out in accordance with the perioperative positioning. Following the identification of the lesion and RLN, the abnormal gland was dissected and removed.

In addition, intraoperative mapping was applied by carbon nanoparticles (CNs) (Chongqing Lummy Pharmaceutical Co. Ltd., China) in both groups. CNs were high lymphatic tropism, when injected into the thyroid gland could immediately black stain the thyroid and the surrounding lymphatic system.^30^, ^35^, ^41^ The strong visual contrast between the parathyroid and the surrounding tissue permitted a more effective and safer dissection.

### 2.6 Surgical outcomes and follow‐up visits

Primary surgical outcomes incorporated postoperative serum levels of calcium and PTH, operation time, estimated blood loss (EBL), conversions, intraoperative morbidities, and postoperative complications. Intraoperative morbidities included vascular and other peripheral organ injuries. Postoperative complications mainly included RLN injury, local seroma, and subcutaneous hematoma. Vocal cord mobility was repeated postoperatively by laryngoscopy in all cases. Neck swelling and tenderness were recorded to assess flap seroma or hematoma.

Secondary outcomes comprised surgical stress and postoperative recovery. Surgical stress response was evaluated through postoperative pain and traumatic index. About 4 h after surgery and on post‐operative day (POD) 1, pain intensity was assessed using a standard visual analogue score (VAS), with a score of 0 to 10 corresponding to no pain to the most severe pain.^42^ Immunologic parameters (IgA, IgG, IgM, and IgE), complements (C3, C4, and CH50), and inflammatory factors, including erythrocyte sedimentation rate (ESR) and C‐reactive protein (CRP) were examined 2 days pre‐operation and 1 day post‐operation. Other recovery parameter included the length of postoperative hospitalization as well.

Follow‐up visit was initiated at 1 month after surgery, with the interval of 3 months. Postoperative recovery, occurrence of complications, serum levels of calcium and PTH were assessed at every visit. The biochemical cure was defined as serum calcium and PTH in the normal range 6 months after surgery. Recurrent hyperparathyroidism was defined as the reappearance of hypercalcemia after 6 months of normal postoperative serum calcium.^5^ All cases were followed more than 1 year.

### 2.7 Measurement of cosmesis

To assess the cosmetic outcomes at the scar proliferation phase (3 months after surgery) and stabilization phase (1 year after surgery), the patient satisfaction score (PSS) and the patient and observer scar assessment scale (POSAS) were employed. The PSS ranges from 1 to 4 with 1 being the most satisfied and 4 the least. The POSAS is made up by the observer scar assessment scale (OSAS) and the patient scar assessment scale (PSAS). There are five determining factors under OSAS, including pigmentation, vascularization, relief, pliability, and thickness. And each aspect is scored by a 10‐point scale, which starts at 1 for normal skin and goes to 10 for the worst scar. The overall score could fall between 5 and 50 points. The PSAS rates scar appearance from six aspects (itchiness, pain, stiffness, color, irregularity and thickness). One to 10 points per section corresponds to normal skin to severe scarring, and the overall score is within the range of 6–60 points.^43^, ^44^, ^45^ The length of incision was documented during the proliferation phase. Moreover, among the TASSEP patients, especially young female patients, breast functionality and the mammographic accuracy were determined during the stabilization phase, together with nipple deformations and sensory abnormalities in the areolar region.

### 2.8 Statistics

The learning curve was established using a cumulative sum (CUSUM) analysis.^46^ For quantitative variables, the results were presented as mean ± SD. Student's *t*‐test was applied to calculate the differences between groups. Pearson's χ2 and Fisher's exact tests were performed to assess categorical variables. Prism version 9.5.0 (GraphPad, California, USA) and SPSS version 26.0 (IBM, New York, USA) were applied for statistical analysis. Results were considered statistically significant at *p* < 0.05.

## 3 RESULTS

### 3.1 Demographics information and surgical planning

A detailed description of the patient characteristics before PSM was presented in Table 1. After matching, TASSEP group and COP group were well balanced, with no significant differences in any baseline demographics (*p* > 0.05, Table 2). The TASSEP group comprised 40 patients: 9 (22.5%) males and 31 (77.5%) females, and the mean age was 48.58 ± 11.17 years. The COP group comprised 40 patients, including 11 (27.5%) males and 29 (72.5%) females, with a mean age of 49.18 ± 13.31 years. Mean preoperative PTH levels of TASSEP and COP groups were well above the normal range, 200.84 ± 208.95 pg/mL and 238.95 ± 186.26 pg/mL, respectively (*p* = 0.464, Table 3). Mean serum levels of calcium and phosphorus were similar between TASSEP and COP groups (2.82 ± 0.32 mmol/L vs. 2.81 ± 0.13 mmol/L, *p* = 0.889; 0.85 ± 0.18 mmol/L vs. 0.82 ± 0.14 mmol/L, *p* = 0.493, Table 3). In TASSEP, symptomatic hyperparathyroidism was found in 32 (80%) patients: 17 patients had renal calculi, 4 had gastrointestinal symptoms, 8 had osteoporosis/osteopenia and 3 had neuromuscular or psychiatric symptoms. In COP, 31 (77.5%) patients presented clinical symptoms related to hyperparathyroidism: 18 had renal calculi, 2 had gastrointestinal symptoms, 7 had osteoporosis/osteopenia and 4 had neuromuscular or psychiatric symptoms.

**TABLE 1:** Baseline characteristics before PSM.

Characteristics	TASSEP (*n* = 40)	COP (*n* = 176)	*p*‐value
Age (mean ± SD, years)	48.58 ± 11.17	57.34 ± 13.96	<0.05
Sex (*n*)			
Male	9	56	0.340
Female	31	120	
BMI (mean ± SD, kg/m^2^)	23.52 ± 3.17	24.42 ± 3.05	0.096
Lesion size (mean ± SD, cm)	1.59 ± 0.60	1.73 ± 0.65	0.209
Lesion location (*n*)			
Right	19	101	0.292
Left	21	75	
Preoperative PTH (mean ± SD, pg/mL)	200.84 ± 208.95	234.17 ± 162.23	0.269
Preoperative calcium (mean ± SD, mmol/L)	2.82 ± 0.32	2.82 ± 0.13	0.835
Preoperative phosphorus (mean ± SD, mmol/L)	0.85 ± 0.18	0.84 ± 0.17	0.785

Abbreviations: BMI, Body Mass Index; COP, conventional open parathyroidectomy; PSM, propensity score matching; TASSEP, trans‐areola single site endoscopic parathyroidectomy.

**TABLE 2:** Characteristics after PSM.

Characteristics	TASSEP (*n* = 40)	COP (*n* = 40)	*p*‐value
Age (mean ± SD, years)	48.58 ± 11.17	49.18 ± 13.31	0.840
Sex (*n*)			
Male	9	11	0.797
Female	31	29	
BMI (mean ± SD, kg/m^2^)	23.52 ± 3.17	23.63 ± 2.82	0.879
Clinical manifestations (*n*)			
Asymptomatic hyperparathyroidism	8	9	>0.999
Symptomatic hyperparathyroidism	32	31	
Renal calculi	17	18	
Gastrointestinal symptoms	4	2	
Osteoporosis/osteopenia	8	7	
Neuromuscular or psychiatric symptoms	3	4	
Lesion size (mean ± SD, cm)	1.59 ± 0.60	1.93 ± 0.84	0.054
Lesion location (*n*)			
Upper right	7	3	0.360
Lower right	12	17	
Upper left	9	6	
Lower left	12	14	
Lesion and thyroid relationship (*n*)			
Type A (compact)	16	19	0.653
Type B (non‐compact)	24	21	

Abbreviations: COP, conventional open parathyroidectomy; TASSEP, trans‐areola single site endoscopic parathyroidectomy.

**TABLE 3:** Surgical profile.

Variables	TASSEP (*n* = 40)	COP (*n* = 40)	*p*‐value
Conversion to open (*n*)	0	^a^	
Intraoperative morbidity (*n*)			
Vascular injury	0	0	1
RLN injury	0	0	1
Operation time (mean ± SD, min)	80.83 ± 11.95	76.95 ± 7.30	0.084
First 10 cases	95.20 ± 12.26	^a^	
The following 10 cases	81.30 ± 8.56	^a^	
Last 20 cases	73.40 ± 4.49	^a^	
Estimated blood loss (mean ± SD, ml)	20.42 ± 10.21	18.86 ± 12.59	0.649
Postoperative hospitalization (mean ± SD, days)	3.65 ± 0.92	3.75 ± 1.14	0.691
PTH (mean ± SD, pg/mL)			
Pre‐operation	200.84 ± 208.95	238.95 ± 186.26	0.464
POD 1	25.77 ± 11.56**	26.29 ± 13.86**	0.876
POD 30	22.40 ± 12.41**	25.22 ± 13.90**	0.418
Calcium (mean ± SD, mmol/L)			
Pre‐operation	2.82 ± 0.32	2.81 ± 0.13	0.889
POD 1	2.32 ± 0.14**	2.32 ± 0.17**	0.818
POD 30	2.28 ± 0.08**	2.29 ± 0.08**	0.608
Phosphorus (mean ± SD, mmol/L)			
Pre‐operation	0.85 ± 0.18	0.82 ± 0.14	0.493
POD 1	1.14 ± 0.27**	1.16 ± 0.15**	0.792
POD 30	1.17 ± 0.17**	1.19 ± 0.13**	0.632

*Note*: Serum PTH, calcium and phosphorus levels on POD 1 and POD 30 were compared with pre‐operative ones in the trans‐areola single site endoscopic parathyroidectomy (TASSEP) and conventional open parathyroidectomy (COP) group.

** *p* < 0.01.

^a^ Means the non‐existed parameters.

Pathological examination of the lesion confirmed the diagnosis of a solitary parathyroid adenoma in all cases. Comparing TASSEP group with COP group, there were no significant differences in mean adenoma size (1.59 ± 0.60 cm vs. 1.93 ± 0.84 cm, *p* = 0.054). Lesion position was obtained by ultrasonography, CT, ^99m^Tc‐sestamibi and 3D virtual imaging, and the preoperative localization was always confirmed during the operation. Of the 40 TASSEPs, 7 upper right, 12 lower right, 9 upper left, and 12 lower left parathyroid adenomas were removed. Of the 40 COPs, 3 adenomas were upper right, 17 were lower right, 6 were upper left, and 14 were lower left. The difference in lesion location was of no statistical significance (*p* = 0.360, Table 2). In view of the 3D visualization model, the relationship between thyroid and parathyroid glands can be classified into type A (compact type) and type B (non‐compact type).^41^ The classification information was also verified during the operation. Sixteen in TASSEP and 19 in COP were type A; 24 TASSEP and 21 COP patients were type B (*p* = 0.653, Table 2).

### 3.2 Surgical profile and postoperative recovery

In all cases parathyroidectomy was completed successfully, the occurrence of intraoperative morbidities, such as vascular and peripheral organ injuries were absent. Of the patients scheduled for TASSEP, no conversion to the traditional open approach was required.

**FIGURE 5:**
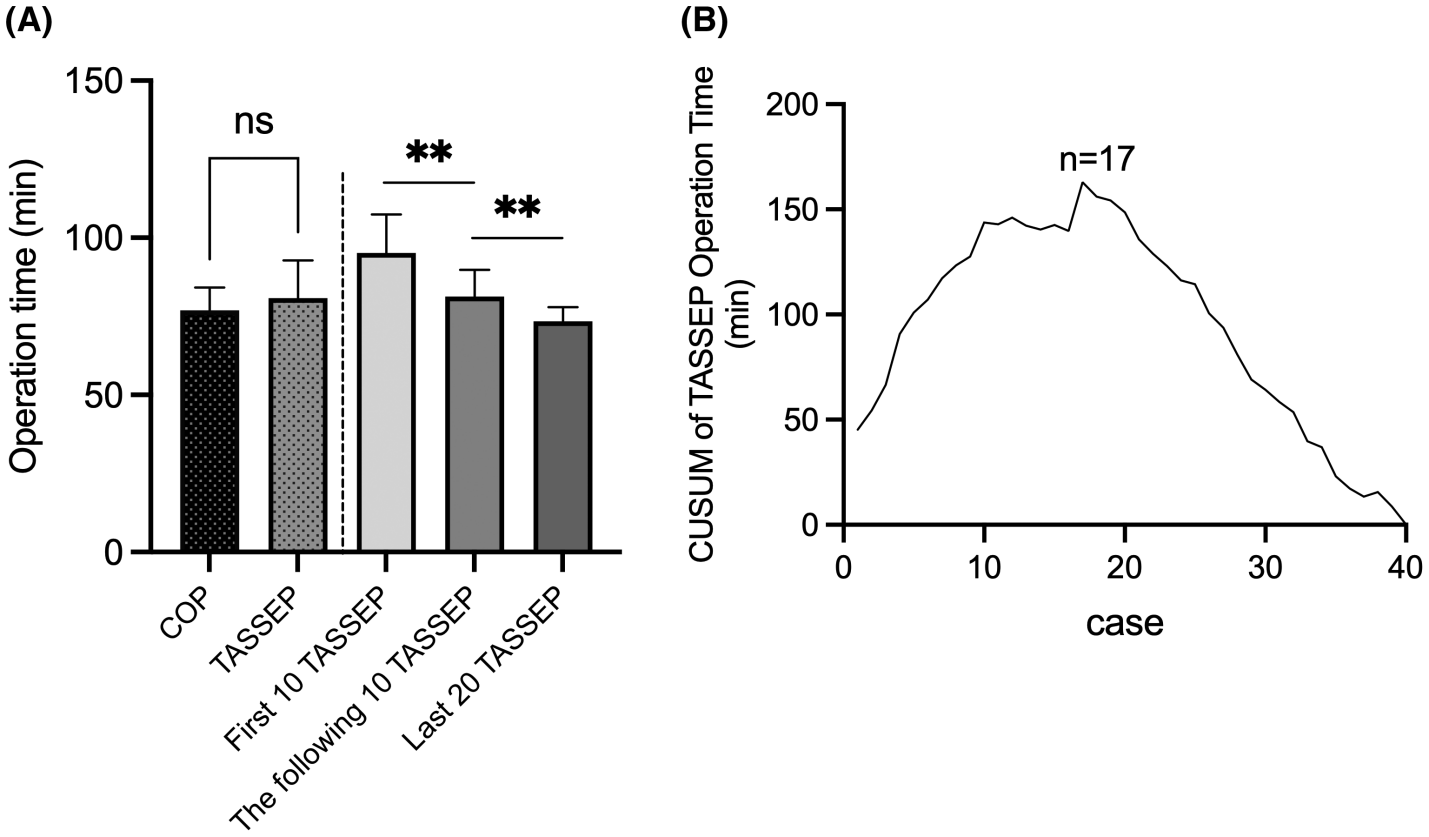
Operation length of trans‐areola single site endoscopic parathyroidectomy (TASSEP) and COP groups. (A) Comparisons of the operation time. The mean operation time of TASSEP and COP was 80.83 ± 11.95 min and 76.95 ± 7.30 min, with no statistical significance (*p* > 0.05). For TASSEP, significant improvements in operation time could be noted over time, with the first 10 cases taking 95.20 ± 12.26 min, the following 10 cases 81.30 ± 8.56 min, and the last 20 cases 73.40 ± 4.49 min (*p* < 0.01). (B) The cumulative sum (CUSUM) curve of TASSEP operation time. With the experience of 17 cases, the surgeon transitioned from the skill acquisition period to the proficiency period. The operation time significantly improved from 90.41 ± 12.34 min to 73.74 ± 4.34 min. ** p < 0.01.

There was no significant difference between TASSEP and COP in terms of operation duration (80.83 ± 11.95 vs. 76.95 ± 7.30 min, *p* = 0.084, Table 3). Based on the accumulated understanding and mastery of TASSEP, significant improvements could be noted in operation time, with the first 10 cases taking 95.20 ± 12.26 min, the following 10 cases 81.30 ± 8.56 min, and the last 20 cases 73.40 ± 4.49 min (*p* < 0.01, Figure 5A). CUSUM analysis was employed to better define the learning curve of TASSEP, and the turning point was identified as case 17 (Figure 5B).^47^ After the end‐point of the learning curve, the operation time of TASSEP significantly improved from 90.41 ± 12.34 min (case 1–17) to 73.74 ± 4.34 min (case 17–40). EBL was evaluated using dry medical gauze strips (0.5 × 5 cm), and did not differ significantly between TASSEP and COP (20.42 ± 10.21 mL vs. 18.86 ± 12.59 mL, *p* = 0.649). And the length of postoperative hospital stay was similar (3.65 ± 0.92 days in TASSEP vs. 3.75 ± 1.14 days in COP, *p* = 0.691).

**FIGURE 6:**
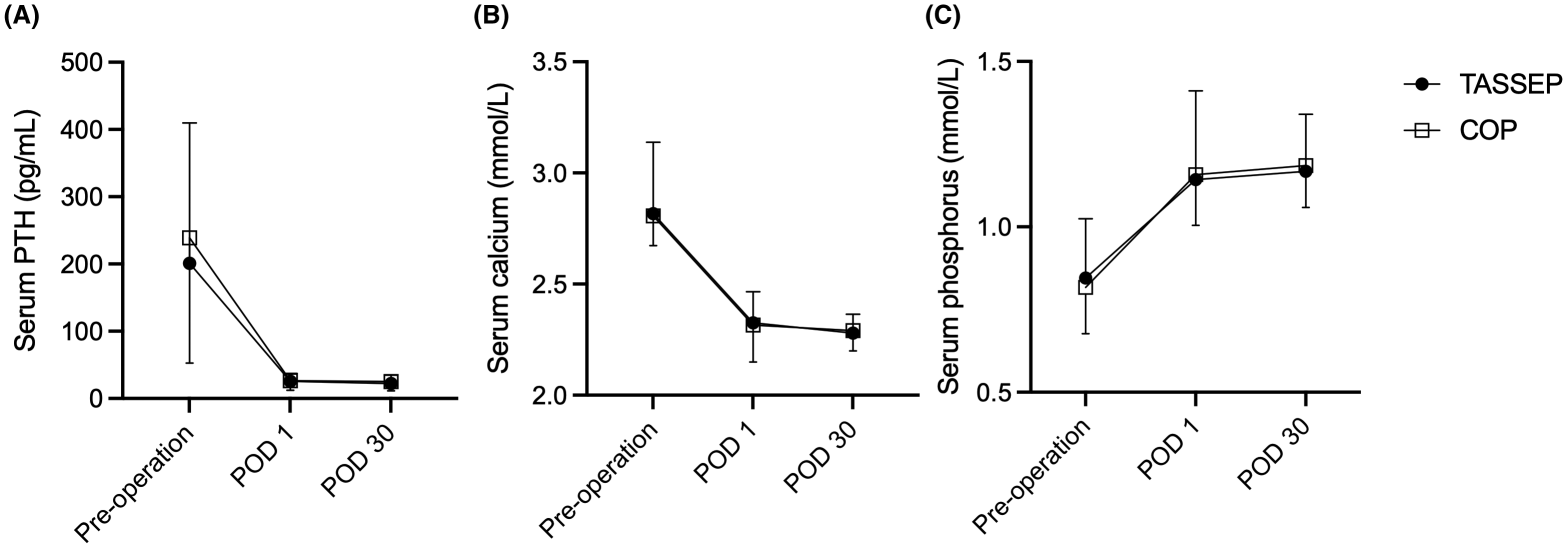
The results of serum PTH, calcium and phosphorus levels in trans‐areola single site endoscopic parathyroidectomy (TASSEP) and COP group. (A) On post‐operative day (POD) 1, mean PTH level dropped from 200.84 ± 208.95 pg/mL to 25.77 ± 11.56 pg/mL in TASSEP, and from 238.95 ± 186.26 pg/mL to 26.29 ± 13.86 pg/mL. (B) On POD 1, mean serum calcium fell to 2.32 ± 0.14 mmol/L in TASSEP, and to 2.32 ± 0.17 mmol/L in COP. (C) On POD 1, mean serum phosphorus recovered to 1.14 ± 0.27 mmol/L in TASSEP, and to 1.16 ± 0.15 mmol/L in COP.

In both groups, a significant decrease was observed in PTH level on POD 1 compared with pre‐operation PTH level (*p* < 0.01). In TASSEP, PTH level on POD 1 decreased to an average of 25.77 ± 11.56 pg/mL compared to 200.84 ± 208.95 pg/mL before operation, while in COP group, POD 1 PTH level dropped from preoperative 238.95 ± 186.26 to 26.29 ± 13.86 pg/mL (Table 3, Figure 6A). In these two groups, postoperative serum calcium and phosphorus were significantly recovered compared with the preoperative period (*p* < 0.01). In TASSEP, the postoperative serum calcium fell to 2.32 ± 0.14 mmol/L, a similar downward trend was observed in COP, with serum calcium falling to 2.32 ± 0.17 mmol/L (Table 3, Figure 6B). As for serum phosphorus, concentrations were significantly elevated after surgery: TASSEP had 1.14 ± 0.27 mmol/L, and COP had 1.16 ± 0.15 mmol/L (Table 3, Figure 6C). There were statistically non‐significant differences in terms of serum levels of calcium, phosphorus and PTH on POD 1 between the groups (*p* > 0.05, Table 3).

The number of complications was low. Two temporary ipsilateral RLN paresis were noted through postoperative laryngoscopy, one for each of the TASSEP and COP group (*p* > 0.999), which were resolved within several weeks. No permanent RLN injuries were noted at 6 months follow‐up. In TASSEP group, no identifiable subcutaneous hematoma or seroma was detected after compressive dressing. Three cases of slight subcutaneous hematoma or seroma were treated conservatively with observation or compression in COP group (*p* = 0.241).

The median follow‐up duration was 23 months (range 12–36 months) for TASSEP and 24 months (range 12–38 months) for COP group. Serum levels of calcium, phosphorus and PTH at 1 month after operation were shown in Table 3, with no significant differences between the two groups (*p* > 0.05, Figure 6). All patients achieved a biochemical cure with normal serum calcium and PTH at the 6‐month follow‐up. During the follow‐up, patients with preoperative symptomatic hyperparathyroidism claimed improvements in symptoms. No recurrent disease was documented.

### 3.3 Surgical stress

Mean postoperative pain, evaluated by VAS system, was scored as 2.50 ± 0.99 in the patients who underwent TASSEP, and 3.15 ± 1.09 among the patients treated with COP on operation day. Additionally, the score was 1.92 ± 0.84 in TASSEP group versus 2.65 ± 1.08 in COP group on POD 1. The differences were both statistically significant (*p* < 0.05). These data indicated an alleviated pain intensity in TASSEP compared with COP (Table 4).

**TABLE 4:** Evaluation of surgical stress.

Variables	TASSEP (*n* = 40)	COP (*n* = 40)	*p*‐value
VAS pain score (mean ± SD)			
Operation day	2.50 ± 0.99	3.15 ± 1.09	0.034
One day after surgery	1.92 ± 0.84	2.65 ± 1.08	0.029
IgE (mean ± SD, IU/mL)			
Before surgery	36.22 ± 25.56	33.58 ± 24.61	0.762
After surgery	32.57 ± 27.03	28.14 ± 20.12	0.886
IgA (mean ± SD, g/L)			
Before surgery	2.15 ± 0.51	2.42 ± 0.49	0.786
After surgery	1.95 ± 0.33	1.89 ± 0.31*	0.833
IgM (mean ± SD, g/L)			
Before surgery	1.37 ± 0.51	1.41 ± 0.56	0.993
After surgery	1.22 ± 0.39	1.17 ± 0.34*	0.884
IgG (mean ± SD, g/L)			
Before surgery	12.20 ± 1.47	12.92 ± 1.65	0.740
After surgery	9.80 ± 1.25*	8.64 ± 1.02*	0.833
C3 (mean ± SD, g/L)			
Before surgery	1.09 ± 0.12	1.24 ± 0.15	0.079
After surgery	1.13 ± 0.17	1.16 ± 0.15	0.118
C4 (mean ± SD, g/L)			
Before surgery	0.27 ± 0.11	0.22 ± 0.04	0.151
After surgery	0.25 ± 0.08	0.23 ± 0.06	0.091
CH50 (mean ± SD, U/mL)			
Before surgery	39.77 ± 9.21	41.56 ± 9.63	0.976
After surgery	36.89 ± 9.10	44.22 ± 10.81	0.487
CRP (mean ± SD, mg/L)			
Before surgery	1.83 ± 1.47	1.65 ± 1.24	0.845
After surgery	6.77 ± 3.35*	21.83 ± 8.57**	< 0.001
ESR (mean ± SD, mm/h)			
Before surgery	7.77 ± 3.83	7.32 ± 3.70	0.143
After surgery	19.20 ± 6.65*	36.44 ± 10.85**	0.002

Abbreviations: COP, conventional open parathyroidectomy; TASSEP, trans‐areola single site endoscopic parathyroidectomy; VAS, Visual Analog Score.

* *p* < 0.05;

** *p* < 0.01.

**FIGURE 7:**
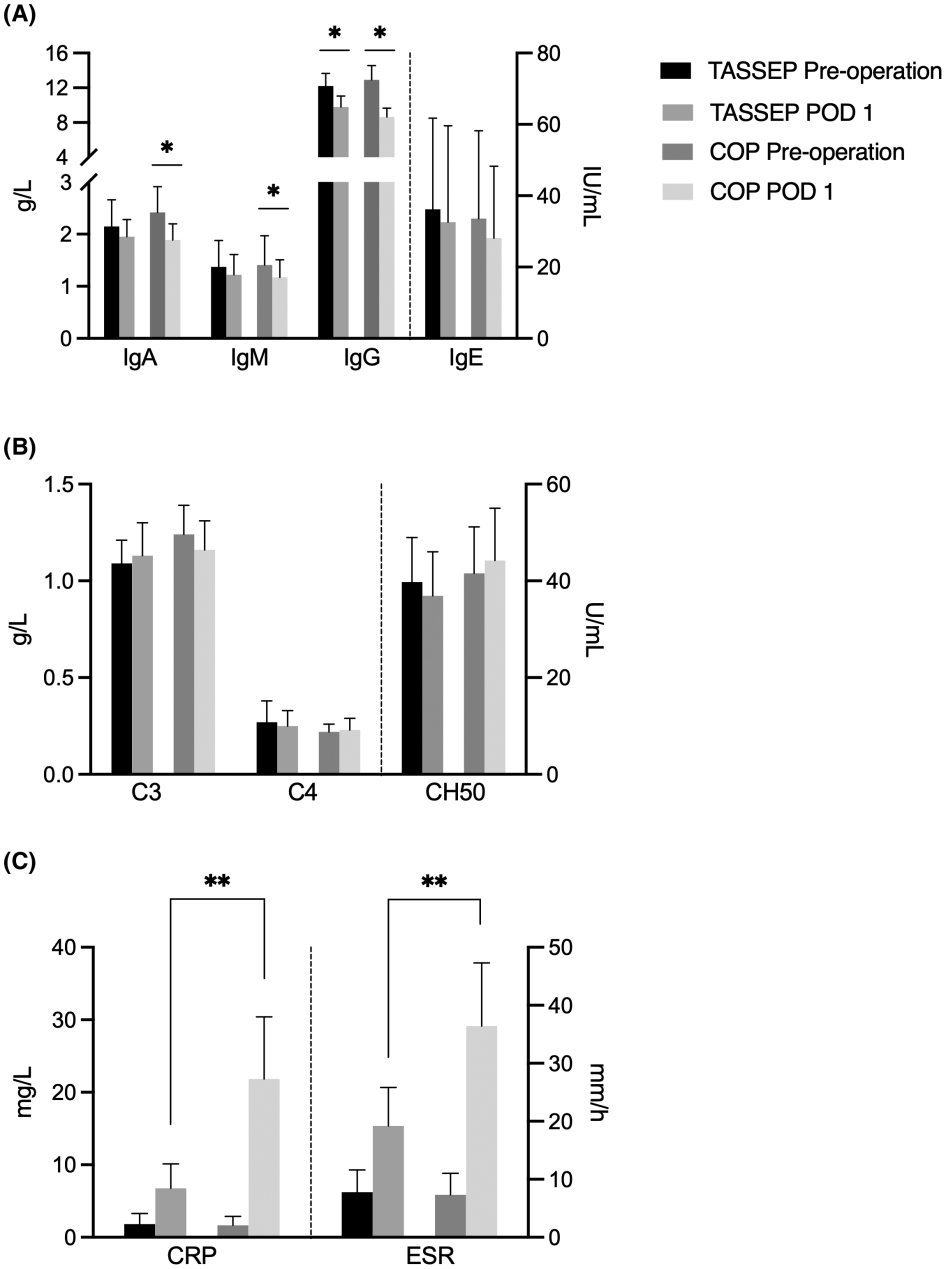
Surgery‐related indicators in trans‐areola single site endoscopic parathyroidectomy (TASSEP) and COP groups. (A) Comparisons of immunological parameters before and after surgery. Downtrends could be observed in both groups. In addition to a significant drop in IgG in both groups after surgery, IgA and IgM also significantly decreased in COP group (*p* < 0.05). (B) Comparisons of complements before and after surgery. (C) Comparisons of traumatic parameters before and after surgery. Elevated CRP and ESR levels in TASSEP were significantly lower than those in COP on POD 1 (*p* < 0.01), which indicated relatively relieved surgical trauma in TASSEP group.

The pre‐ and post‐operative states were evaluated within either group. In TASSEP, IgA, IgE, IgM, C3, C4, CH50 did not alter significantly; whereas IgG significantly decreased after surgery (12.20 ± 1.47 vs. 9.80 ± 1.25 g/L, *p* < 0.05, Figure 7A, Figure 7B); CRP and ESR increased (1.83 ± 1.47 vs. 6.77 ± 3.35 mg/L, *p* < 0.05; 7.77 ± 3.83 vs. 19.20 ± 6.65 mm/h, *p* < 0.05, Figure 7C). In COP, besides the same tendencies of IgG, CRP and ESR as those in TASSEP, IgA and IgM also significantly decreased (*p* < 0.05). Remaining IgE, C3, C4, and CH50 showed no difference before and after surgery (Table 4, Figure 7).

First, as shown in Table 4, there were no differences in CRP, ESR, IgG, IgA, IgE, IgM, and C3, C4, CH50 between the two groups before surgery. The comparable pre‐surgical stress and immunological status allowed further comparison of the surgical burden between the two groups. However, on POD 1, traumatic parameters (CRP and ESR) in TASSEP were significantly lower than those in COP (6.77 ± 3.35 vs. 21.83 ± 8.57 mg/L, *p* < 0.001; 19.20 ± 6.65 vs. 36.44 ± 10.85 mm/h, *p* = 0.002), whereas remaining immunological parameters and complements did not show any difference. Overall, although TASSEP did create surgical burden and immunological repression, COP might provoke more damaging traumatic stress and immunological repression.

### 3.4 Cosmetic results

Table 5 showed the cosmesis‐related results of the comprehensive comparison between the two groups. In TASSEP, the mean incision was 1.85 ± 0.16 cm, while the cervical incision with a mean length of 4.90 ± 0.20 cm was quite eye‐catching in COP (*p* < 0.001). The incisions in TASSEP were almost invisible at 3‐month postoperative follow‐up visit (Figure 1D). Among young female patients who underwent TASSEP, breast function and mammographic accuracy were unchanged. Additionally, neither nipple deformity nor impaired areolar sensation was documented.

**TABLE 5:** Cosmetic results.

Variables	TASSEP (*n* = 40)	COP (*n* = 40)	*p*‐value
Incision length (mean ± SD, cm)	1.85 ± 0.16	4.90 ± 0.20	<0.001
PSS (mean ± SD)			
Proliferation stage	1.77 ± 0.59	3.20 ± 0.77	<0.001
Stabilization stage	1.31 ± 0.47	2.55 ± 0.76	<0.001
POSAS (mean ± SD)			
OSAS			
Proliferation stage	11.60 ± 3.62	24.91 ± 8.33	<0.001
Stabilization stage	9.54 ± 2.79	20.05 ± 6.34	<0.001
PSAS			
Proliferation stage	17.62 ± 4.96	31.90 ± 9.76	<0.001
Stabilization stage	13.15 ± 4.81	24.70 ± 10.13	<0.001

Abbreviations: COP, conventional open parathyroidectomy; OSAS, observer scar assessment scale; POSAS, patient and observer scar assessment scale; PSAS, patient scar assessment scale; PSS, patient satisfaction score; TASSEP, trans‐areola single site endoscopic parathyroidectomy.

As to cosmetic satisfaction evaluation, TASSEP patients scored significantly lower on PSS than open counterpart at proliferation period (1.77 ± 0.59 vs. 3.20 ± 0.77, *p* < 0.001) and stabilization period (1.31 ± 0.47 vs. 2.55 ± 0.76, *p* < 0.001), respectively. Concerning scar evaluation, the mean summarized score of OSAS was 11.60 ± 3.62 versus 24.91 ± 8.33 between TASSEP and COP groups at proliferation phase (*p* < 0.001), and 9.54 ± 2.79 versus 20.05 ± 6.34 at stabilization phase (*p* < 0.001), respectively. Whereas, the mean summarized score of PSAS was 17.62 ± 4.96 and 31.90 ± 9.76 for TASSEP and COP groups at proliferation stage (*p* < 0.001), and 13.15 ± 4.81 versus 24.70 ± 10.13 at stabilization period, respectively (*p* < 0.001). The above results revealed that both groups, especially in TASSEP group, achieved a satisfied and enhanced cosmetic effect during the recovery stage. In general, TASSEP provided significant cosmetic and functional advantages in comparison to open surgery.

## 4 DISCUSSION

Surgery for PHPT has evolved from bilateral neck exploration to focused exploration based on precise preoperative localization.^8^ However, there exists the unfixed anatomical relationship between the parathyroid and thyroid gland, which makes the focused and accurate preoperative localization a challenge.^24^, ^48^ Moreover, for indiscernible lesions, conventional imaging with only 2D visualization could not fully clarify the spatial variations of anatomical surroundings, which limited precise surgical planning, and for endoscopic procedures increased the likelihood of conversion to open surgery.^22^, ^49^, ^50^ To date, with the development of interactive technology, 3D imaging and visualization technology has already shown its superiority in preoperative planning and intraoperative navigation in many surgical fields.^51^, ^52^, ^53^, ^54^, ^55^ The highly detailed, augmented realistic images provided by 3D digitization assisted in individualized and accurate surgical planning, such as peripheral anatomy demonstration, tissue separation procedure, and surgical ligation sequence. Our study confirmed 3D demonstration with anatomical details could portrait anatomical variations, identify lesion boundary and its spatial relationship with adjacent tissues more clearly, which directed surgery toward the specific target, and allowed for precise resection. Under the magnification of the endoscopic approach, the parathyroid adenoma was usually readily located, and the RLN was also well recognized. By intraoperative CNs mapping, the indiscernible lesions could be better demonstrated, with black staining of thyroid helping identify compact parathyroid glands, and central lymph node staining helping recognize non‐compact glands.^41^

As to endoscopic parathyroidectomy and thyroidectomy, the main conflict is how to balance the extra surgical burden caused by subcutaneous tissues dissection with the cosmetic results. Recently, advances in single‐port endoscopic techniques have relieved surgical burden with ideal oncological radiation.^56^, ^57^, ^58^ Therefore, with precise preoperative localization, endoscopic parathyroidectomy via multiple‐site approach might be unnecessary, and a single‐site approach was put forward to further minimize surgical burden. In our study, all TASSEPs were performed successfully, and serum calcium and PTH levels remained normal throughout the follow‐up period in TASSEP and COP groups. To conclude, surgical results comparable to those of open surgery can be obtained through one trans‐areola incision without altering breast function and mammographic screening accuracy especially in young female patients. Evidenced by lower postoperative CRP and ESR levels compared with COP, TASSEP was proposed via one subcutaneous tunnel to minimize the dissection area and collateral injury, which could help alleviate surgical burden. Meanwhile, lower postoperative pain assessed by VAS also implied a clinical significance of the alleviated pain intensity in the TASSEP group. The single‐site entry was strategically placed around the border of areola, which could be completely concealed within the areola area in the stabilization phase. During the follow‐up period, TASSEP patients exhibited significantly higher cosmetic satisfaction which may explained by the absence of a collar incision. In addition, neither nipple deformation nor the influenced sensation of areola area was noticed.

Although with evidence pointing to better cosmesis, alleviated surgical burden, and similar excellent cure rate, the technological barriers and steep learning curve of TASSEP had kept it from further application. To address these issues, the piercing needle retractors and the mini‐clamps were introduced to accomplish a series of surgical maneuvers such as pull, push, suspension, and compression, which could create a triangular space with the strap muscles retracting. With the elasticity and force feedback, the mini‐clamp was positioned at the level of the thyroid cartilage, enabling the involved tissues to be grasped and lifted superiorly. The surrounding structures could then be anatomically isolated and suspended to expose surgical area. The lesion could be precisely detached and separated with the scalpel. Intraoperative bleeding was considered a major challenging technique as suction and hemostasis could not be achieved with only one apparatus at a time. In our series, the harmonic scalpel could effectively stop bleeding in cases where the mini‐clamps assisted in compressing and grasping the involved area for better visual exposure. The increased flexibility provided by needle retractors and mini‐clamps helps reduce the operational difficulty and the recognition time of this procedure. In current study, 17 cases were required to qualify for the initial surgical proficiency stage in TASSEP. Whereas the end point of the learning curve in trans‐areola single site endoscopic thyroidectomy (TASSET) was case 41, which indicated that the surgeons could adopt TASSEP more quickly than TASSET, making it a promising alternative to open surgery for both surgeons and patients.^47^ Therefore, in line with the standardized TASSEP procedure, technique challenges could be overcome readily with reliable operational precision and safety.

TASSEP offers a feasible treatment for solitary parathyroid adenoma with an excellent success rate and a minimal complication rate similar to open surgery. When introducing TASSEP into the clinical realm, for individual patients, careful selection is paramount, and precise procedures should be tailored. With progress of experience and more understanding of this technique, eligibility criteria can be broadened and, when necessary, bilateral neck exploration can be fulfilled. Furthermore, the current study was limited with the retrospective experimental design. Thus, the propensity score matching method was implemented to attenuate the potential bias. To further validate our findings, prospective and multicenter studies with larger sample‐size are warranted.

## 5 CONCLUSION

Based on precise preoperative localization and intraoperative planning provided by 3D modeling, TASSEP can be feasibly performed for well‐selected patients. Along with proven clinical outcomes comparable to those of open surgery, TASSEP can yield superior cosmetic results and induce a somewhat milder surgical burden.

## AUTHOR CONTRIBUTIONS

**Ling Zhan:** Conceptualization (lead); data curation (lead); formal analysis (lead); validation (lead); writing – original draft (lead). **Hao Ding:** Conceptualization (supporting); data curation (supporting); formal analysis (supporting); supervision (lead). **Qiwu Zhao:** Investigation (supporting); project administration (supporting); writing – review and editing (supporting). **Jinyue Liu:** Data curation (supporting); formal analysis (supporting); writing – review and editing (lead). **Juyong Liang:** Investigation (supporting). **Ming Xuan:** Investigation (supporting); project administration (supporting). **Jie Kuang:** Investigation (supporting); methodology (supporting); project administration (supporting). **Jiqi Yan:** Investigation (supporting); methodology (supporting); project administration (supporting). **Lingxie Chen:** Supervision (supporting); validation (supporting); writing – review and editing (supporting). **Wei Cai:** Supervision (supporting); validation (supporting); visualization (supporting). **Weihua Qiu:** Conceptualization (lead); funding acquisition (lead); supervision (lead); visualization (lead); writing – review and editing (lead).

## FUNDING INFORMATION

This study was supported by the National Natural Science Foundation of China (NSFC, 81772558, 82072948).

## CONFLICT OF INTEREST STATEMENT

All authors have no conflicts of interest or financial ties.

## Data Availability

Data available on request from the authors.
